# Genome-Wide Association Analysis Dissects the Genetic Basis of the Grain Carbon and Nitrogen Contents in Milled Rice

**DOI:** 10.1186/s12284-019-0362-2

**Published:** 2019-12-30

**Authors:** Liang Tang, Fan Zhang, Anjin Liu, Jian Sun, Song Mei, Xin Wang, Zhongyuan Liu, Wanying Liu, Qing Lu, Shuangjie Chen

**Affiliations:** 1 Rice Research Institute, Shenyang Agricultural University, Shenyang, 110866 China; 2 Institute of Crop Sciences/National Key Facility for Crop Gene Resources and Genetic Improvement, Chinese Academy of Agricultural Sciences, 12 South Zhong-Guan-Cun Street, Haidian District, Beijing, 100081 China

**Keywords:** Grain nitrogen content, Grain carbon content, Germplasm, Genome-wide association study (GWAS), Rice (*Oryza sativa* L.), Haplotype analysis

## Abstract

**Background:**

Carbon (C) and nitrogen (N) are two fundamental components of starch and protein, which are important determinants of grain yield and quality. The food preferences of consumers and the expected end-use of grains in different rice-growing regions require diverse varieties that differ in terms of the grain N content (GNC) and grain C content (GCC) of milled rice. Thus, it is important that quantitative trait loci (QTLs)/genes with large effects on the variation of GNC and GCC are identified in breeding programs.

**Results:**

To dissect the genetic basis of the variation of GNC and GCC in rice, the Dumas combustion method was used to analyze 751 diverse accessions regarding the GNC, GCC, and C/N ratio of the milled grains. The GCC and GNC differed significantly among the rice subgroups, especially between *Xian*/*Indica* (*XI*) and *Geng*/*Japonica* (*GJ*). Interestingly, in the *GJ* subgroup, the GNC was significantly lower in modern varieties (MV) than in landraces (LAN). In the *XI* subgroup, the GCC was significantly higher in MV than in LAN. One, six, and nine QTLs, with 55 suggestively associated single nucleotide polymorphisms, were detected for the GNC, GCC, and C/N ratio in three panels during a single-locus genome-wide association study (GWAS). Three of these QTLs were also identified in a multi-locus GWAS. We screened 113 candidate genes in the 16 QTLs in gene-based haplotype analyses. Among these candidate genes, *LOC_Os01g06240* at *qNC-1.1*, *LOC_Os05g33300* at *qCC-5.1*, *LOC_Os01g04360* at *qCN-1.1*, and *LOC_Os05g43880* at *qCN-5.2* may partially explain the significant differences between the LAN and MV. These candidate genes should be cloned and may be useful for molecular breeding to rapidly improve the GNC, GCC, and C/N ratio of rice.

**Conclusions:**

Our findings represent valuable information regarding the genetic basis of the GNC and GCC and may be relevant for enhancing the application of favorable haplotypes of candidate genes for the molecular breeding of new rice varieties with specific grain N and C contents.

## Background

As a staple food for more than half of the global population, rice is one of the most widely grown cereals worldwide and is critical for food security. Additionally, it is the source of about 25–50% of the daily protein intake of humans in developing countries, especially in Asia (Deng et al. 2019). Carbon (C) and nitrogen (N) are two fundamental components of starch and protein, which are important determinants of grain yield and quality that influence the milling, appearance, eating and cooking qualities, nutritional qualities, and health benefits of grains (Martin and Fitzgerald 2002). The C/N ratio, which reflects the relative strength of C and N metabolism, is useful for evaluating the metabolic balance between C and N and the growth vigor in crop plants (Xu et al. 2018). Starch and protein account for 70–80 and 7–10% of the components in the rice endosperm, respectively. The synthesis of starch from sugars (or other carbohydrates) requires less energy than the production of other substances in the rice grain, and is conducive to dry-matter accumulation and high yield. The amino acid content in rice is relatively balanced compared with that of other crops, including wheat and maize (Peng et al. 2014). Thus, increasing the grain N content (GNC), grain C content (GCC), and C/N ratio in milled rice is very important for improving the rice nutritional quality and yield. The economic development in Asia, with China as an example, has altered the rice breeding strategy from blindly pursuing higher yield to paying equal attention to high yield and quality as well as decreasing production costs, but maintaining safety (Tang et al. 2017).

Amylose and amylopectin are two types of glucan polymers in starch that are synthesized via the synergistic effects of several enzymes (Jeon et al. 2010). Amylose is mainly composed of a linear chain of alpha-1,4-linked glucose residues, and is synthesized in a reaction catalyzed by the *Wx*-encoded granule-bound starch synthase I (Jeon et al. 2010). Genes at other loci, such as the *du* loci, that are under monogenic recessive control have an additive effect on lowering the amylose content (Yano et al. 1988). Amylopectin has a multiple-cluster structure comprising a highly branched glucan with alpha-1,6-glycosidic bonds, and its synthesis is coordinately catalyzed by the following three classes of enzymes: soluble starch synthases (SSs: SSI, SSIIa, and SSIIIa), starch branching enzymes (BEs: BEI, BEIIa, and BEIIb), and starch debranching enzymes (ISA1 and PUL) (Jeon et al. 2010). The *OsSSI* (*SSS1*) gene encodes starch synthase I, which affects the amylopectin structure, but has no significant effect on the amylose content (Kawakatsu et al. 2010b). The *OsSSIIIa* (*Flo5*) gene encodes starch synthase IIIa, which affects the amylopectin structure, amylose content, and physicochemical properties of rice grain starch (Zhou et al. 2016). Moreover, *OsBEIIb* encodes an amylase starch branching enzyme (SBE IIb) that influences the starch structure in rice endosperm (Lu and Park 2012; Yang et al. 2012).

Rice grain proteins can be categorized as functional proteins (approximately 10%) and seed storage proteins (SSPs; approximately 90%) (Yang et al. 2019). On the basis of solubility-linked physical properties, SSPs comprise the following four protein fractions: albumins, globulins, prolamins, and glutelins (Kawakatsu et al. 2008; Saito et al. 2012). Among these proteins, glutelins are the most abundant, accounting for about 60–80% of all SSPs (Makoto et al. 2003). Because of a higher lysine content and greater digestibility, the nutritional value of rice glutelin is superior to that of other rice storage proteins. Glutelins can be further divided into four groups (GluA, GluB, GluC, and GluD) based on their amino acid sequence similarity (Kawakatsu and Takaiwa 2010). Several glutelin genes have been cloned, including *GluA*, *GluB1*, *GluB6*, *GluB7*, *GluD*, and *OsGZF1* (Kawakatsu et al. 2010a ; Wu et al. 2010; Yi et al. 2014). Additionally, considerable effort has been made toward dissecting the genetic mechanism underlying the rice grain protein content (GPC) (Ren et al. 2014; Terao and Hirose 2015; Tian et al. 2013; Wang et al. 2011; Wang et al. 2010a, b). However, the mechanism mediating the GPC differences remains relatively uncharacterized (Yang et al. 2019). The SSPs are controlled by complex multigene families (Tian et al. 2009). Interestingly, some of these genes not only regulate starch storage, but also affect the protein in the endosperm (She et al. 2010; Wang et al. 2011). The combined effects of *OsAGPL2* and *OsAGPS2b* are very important for the accumulation of storage substances, such as starch and protein, in the rice endosperm (Tang et al. 2016).

Most of the above-mentioned isolated genes affecting the GPC and the grain starch content (GSC) were identified based on various rice mutants. Therefore, a few favorable alleles of these genes have been mined for rice breeding. Although many quantitative trait loci (QTLs) related to the GPC and GSC have been detected by linkage mapping (Cheng et al. 2013; Yao et al. 2017; Ye et al. 2010; Zhang et al. 2008; Zheng et al. 2011) and association studies (Chen et al. 2018; Wang et al. 2017; Xu et al. 2016), most of these studies focused on the crude protein and starch contents or the individual protein and starch fractions in milled rice. There have been no studies on the genetic mechanism controlling the GNC, GCC, and C/N ratio in milled rice.

In this study, a diverse panel consisting of 751 accessions from the 3000 Rice Genomes Project (3K RGP) (3K RGP 2014) was evaluated regarding the GNC and GCC in milled rice to identify related candidate genes in a genome-wide association study (GWAS) with high-density single nucleotide polymorphisms (SNPs). This was followed by a gene-based haplotype analysis. The objectives of our study were as follows: (1) screen representative resources with a distinct GNC, GCC, and C/N ratio in rice germplasm; (2) identify loci and candidate genes associated with the GNC, GCC, and C/N ratio; and (3) mine the favorable haplotypes/alleles of some important candidate genes in rice germplasm.

## Results

### Phenotypic Variations and Correlations

**Fig. 1:**
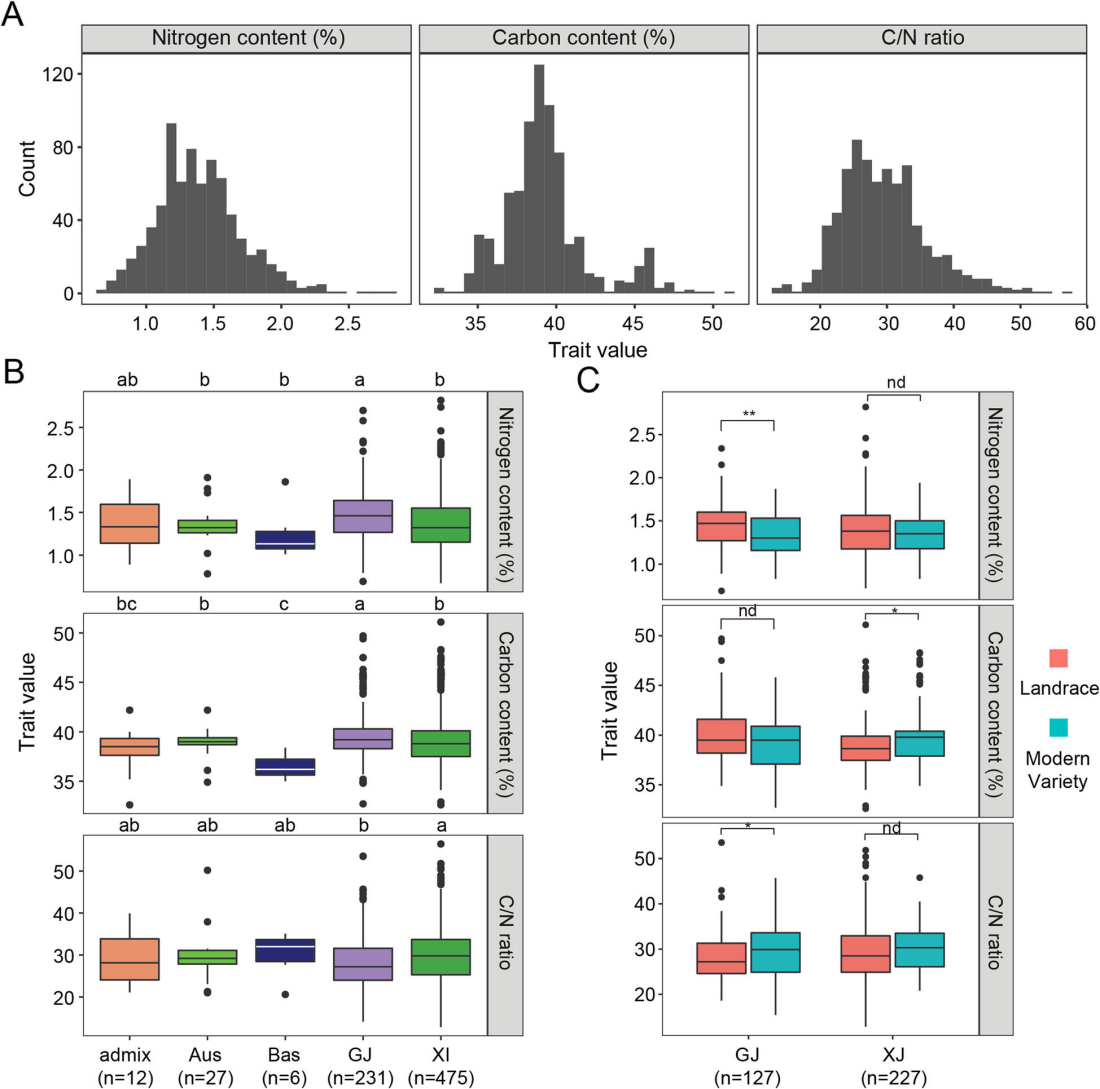
Grain nitrogen content (GNC), grain carbon content (GCC), and the C/N ratio of milled rice and correlations among these traits in rice subgroups. **a** Phenotypic distribution of the GNC, GCC, and C/N ratio in the whole population. **b** The GNC, GCC, and C/N ratio in five rice subgroups. Different letters above the boxplots indicate significant differences among subgroups (*P* < 0.05) based on Duncan’s test. **c** Differences in the GNC, GCC, and C/N ratio between landraces and modern varieties in the *GJ* and *XI* subgroups. *, **, and nd indicate significant differences at *P* < 0.05, *P* < 0.01, and no significant difference, respectively (Student’s *t*-test)

A diverse global collection of 751 *Oryza sativa* L. accessions were evaluated regarding their GNC, GCC, and calculated C/N ratios (Additional file 1: Table S1). A broad phenotypic distribution among the diverse rice accessions from the 3K RGP implied that substantial genetic variations controlled these three traits (Fig. 1a). The average GNC, GCC, and C/N ratio were 1.40% (0.67–2.82%), 39.3% (32.6–51.1%), and 29.5% (12.9–56.4%), respectively. An analysis of five rice subgroups revealed significant differences in these three traits, especially in the *Xian*/*Indica* (*XI*) and *Geng*/*Japonica* (*GJ*) comparisons (Fig. 1b). Specifically, the GNC (mean 1.47%; 0.69–2.70%) and GCC (mean 39.6%; 32.7–49.7%) of *GJ* were higher than the GNC (mean 1.36%; 0.67–2.82%) and GCC (mean 39.2%; 32.6–51.1%) of *XI*. In contrast, the C/N ratio of *GJ* (mean 28.0%; 14.2–53.5%) was lower than that of *XI* (mean 30.2%; 12.9–56.4%) (Fig. 1b and Additional file 1: Table S1). Additionally, the mean values of these three traits (GNC 1.35%, GCC 38.9%, and C/N ratio 29.6%) for *Aus* were similar to those of *XI*, which is consistent with their close phylogenetic relationship (Wang et al. 2018). However, significant differences in the GNC and GCC were observed between the basmati (*Bas*) and *GJ* subgroups, which also have a close phylogenetic relationship (Fig. 1b). Interestingly, in the *GJ* subgroup, the mean GNC (1.38%) of modern varieties (MV) was significantly lower than that (1.48%) of landraces (LAN), whereas there were no significant differences in the mean GNC between the *XI* MV and LAN (Fig. 1c). Regarding the GCC, in the *XI* subgroup, the mean value was significantly higher in the MV (39.9%) than in the LAN (39.1%). In contrast, there was no significant difference in the GCC of the *GJ* MV and LAN. These results suggest the GNC and GCC were affected by intense selective breeding for diverse targets in *GJ* and *XI*. A significant positive correlation between GNC and GCC was determined for the whole population (*r* = 0.30, *P* < 0.001), but the correlation was greater in *XI* (*r* = 0.33, *P* < 0.001) than in *GJ* (*r* = 0.22, *P* < 0.001), suggesting some differentiation in the GNC and GCC between the two rice subgroups (Additional file 2: Table S2). Moreover, significantly negative correlations between GNC and the C/N ratio and no correlations between GCC and the C/N ratio, respectively, were detected for the whole, *XI*, and *GJ* populations (Additional file 2: Table S2). These findings imply that the genetic basis of the GNC and GCC probably differs between the *XI* and *GJ* accessions.

### Single-Locus GWAS for the GNC, GCC, and C/N Ratio

**Fig. 2:**
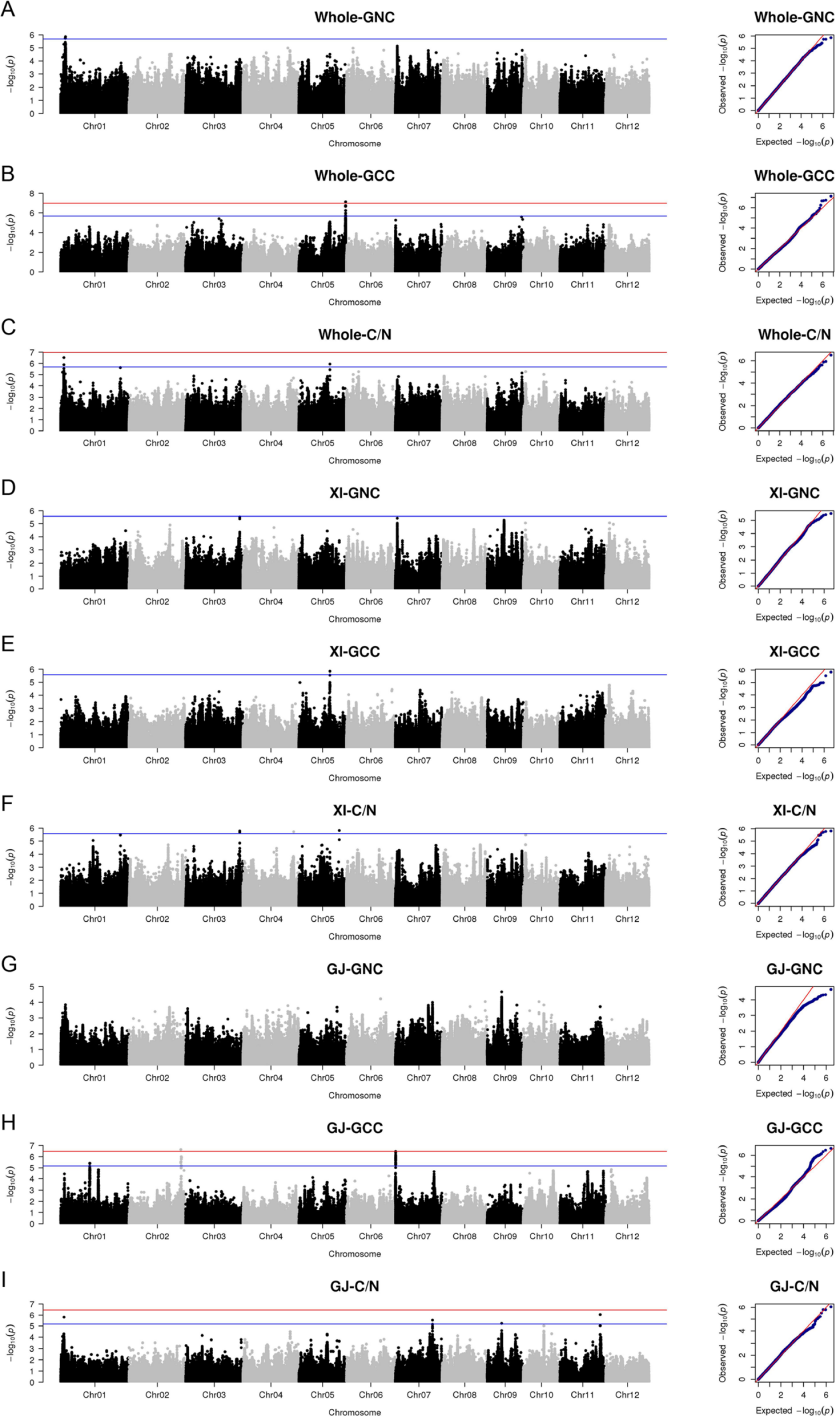
Manhattan and quantile-quantile plots for the single-locus GWAS. **a** Grain nitrogen content (NC), (**b**) grain carbon content (CC), and (**c**) the C/N ratio for the whole GWAS panel. **d** NC, (**e**) CC, and (**f**) the C/N ratio for the *XI* GWAS panel. **g** NC, (**h**) CC, and (**i**) the C/N ratio for the *GJ* GWAS panel. The points in the Manhattan plots indicate the −log_10_(*P*) value. The horizontal red and blue lines indicate the significant and suggestive thresholds calculated as follows: 0.05 and 1 divided by the effective number of independent markers in the GWAS panel, respectively

We conducted a single-locus GWAS to identify loci associated with the GNC, GCC, and C/N ratio in three panels (whole population, *XI*, and *GJ*) (Fig. 2). A total of 2,994,907, 2,118,326, and 1,318,493 filtered SNPs for the whole population, *XI*, and *GJ* panels, respectively, were used for the association analyses with the LMM of EMMAX (Kang et al. 2010). On the basis of a significant 1/N (N indicating the effective number of independent SNPs) calculated with the GEC software (Li et al. 2012), the Bonferroni-corrected genome-wide *P*-value thresholds of 2.09 × 10^− 6^, 2.66 × 10^− 6^, and 6.79 × 10^− 6^ were considered to reflect suggestive associations in the whole population, *XI*, and *GJ* panels, respectively (Additional file 3: Table S3). A total of 55 associated SNPs were detected on chromosomes 1, 2, 3, 4, 5, 7, 9, and 11 in all three panels, including 3, 40, and 12 SNPs associated with the GNC, GCC, and C/N ratio, respectively. These SNPs were located within or neighboring 38 annotated genes in the Nipponbare reference genome IRGSP 1.0 (Additional file 4: Table S4). We combined adjacent significantly associated SNPs within a linkage disequilibrium (LD) block as a QTL associated with the analyzed traits. Specifically, one (*qNC-1.1*), six (*qCC-1.1*, *qCC-2.1*, *qCC-2.2*, *qCC-5.1*, *qCC-5.2*, and *qCC-7.1*), and nine (*qCN-1.1*, *qCN-1.2*, *qCN-3.1*, *qCN-4.1*, *qCN-5.1*, *qCN-5.2*, *qCN-7.1*, *qCN-9.1*, and *qCN-11.1*) QTLs were detected for the GNC, GCC, and C/N ratio, respectively, in all panels (Table 1).

**Table 1:** Sixteen QTLs for the GNC, GCC, and C/N ratio of milled rice identified in a single-locus GWAS

Traits	QTL	Chromosome	Start (bp)	End (bp)	No. of significant SNP	Lead SNP	*P* value of lead SNP	Lead SNP located/flanking genes	Detected panels^a^	Detected panels by multiple-locus GWAS	Known related genes
GNC	*qNC-1.1*	1	2,931,216	2,967,102	3	rs1_2956867	1.38E-06	LOC_Os01g06210-LOC_Os01g06220	*Whole*		
GCC	*qCC-1.1*	1	18,333,711	18,405,691	1	rs1_18390016	3.95E-06	LOC_Os01g33410	*GJ*		
	*qCC-2.1*	2	32,773,074	32,956,207	3	rs2_32881022	2.32E-07	LOC_Os02g53710-LOC_Os02g53720	*GJ*		
	*qCC-2.2*	2	33,160,172	33,274,475	4	rs2_33,160,172	1.00E-06	LOC_Os02g54120	*GJ*		
	*qCC-5.1*	5	19,481,277	19,681,277	1	rs5_19566944	1.44E-06	LOC_Os05g33340	*XI*	*Whole*	*OsPPDKB*
	*qCC-5.2*	5	29,438,881	29,580,663	8	rs5_29562689	7.43E-08	LOC_Os05g51550-LOC_Os05g51560	*Whole*		*NRR; CRCT*
	*qCC-7.1*	7	127,781	283,670	23	rs7_144536	3.42E-07	LOC_Os07g01240	*GJ*		
C/N ratio	*qCN-1.1*	1	1,942,481	1,952,710	2	rs1_1952292	3.05E-07	LOC_Os01g04380	*Whole*		
	*qCN-1.2*	1	1,959,903	2,123,819	2	rs1_2023817	1.57E-06	LOC_Os01g04540	*GJ*		
	*qCN-3.1*	3	34,300,375	34,309,240	2	rs3_34300823	1.66E-06	LOC_Os03g60310-LOC_Os03g60340	*XI*		
	*qCN-4.1*	4	31,985,258	32,185,258	1	rs4_32085258	1.93E-06	LOC_Os04g53850	*XI*		
	*qCN-5.1*	5	19,481,277	19,681,277	1	rs5_19565967	1.14E-06	LOC_Os05g33340	*Whole*		*OsPPDKB*
	*qCN-5.2*	5	25,421,042	25,621,042	1	rs5_25521042	1.52E-06	LOC_Os05g43880-LOC_Os05g43890	*XI*	*XI*	
	*qCN-7.1*	7	23,515,915	23,515,983	1	rs7_23515974	2.99E-06	LOC_Os07g39280	*GJ*		*OsBZR1*
	*qCN-9.1*	9	9,221,280	9,230,703	1	rs9_9222530	5.93E-06	LOC_Os09g15190-LOC_Os09g15200	*GJ*		
	*qCN-11.1*	11	25,603,546	25,604,997	1	rs11_25,603,546	9.05E-07	LOC_Os11g42510-LOC_Os11g42520	*GJ*	*GJ*	

^a^*XI* and *GJ* represent *Xian*/*Indica* and *Geng*/*Japonica*, respectively

### Multi-Locus GWAS for the GNC, GCC, and C/N Ratio

In general, multiple testing correction methods, such as the Bonferroni correction method, for modifying the significant threshold value to control the false positive rate in a single-locus GWAS are so conservative that some associated SNPs may be eliminated. Therefore, we conducted a multi-locus association analysis with the mrMLM algorithm to solve this problem. This multi-locus GWAS detected more loci underlying the GNC, GCC, and C/N ratio than the single-locus GWAS for the same three panels. We identified 130 significant SNPs on all 12 chromosomes in at least one of the three panels, including 45, 34, and 51 SNPs for the GNC, GCC, and C/N ratio, respectively (Additional file 5: Table S5). For the GNC, 18, 27, and 4 SNPs were identified in the whole population, *XI*, and *GJ* panels, respectively, with the SNPs explaining 1.58–6.55%, 0.87–6.99%, and 7.00–14.79% of the phenotypic variations (PVE), respectively (Additional file 5: Table S5). Among these SNPs, four (rs4_1971938, rs4_30789977, rs6_23599588, and rs7_26720430) were detected in both the whole population and *XI* panels. Regarding the GCC, 11, 13, and 10 SNPs on all chromosomes, except for chromosome 10, were detected in the whole population, *XI*, and *GJ* panels, respectively, with PVE values of 1.34–5.78%, 2.43–5.20%, and 2.93–16.49%, respectively. For the C/N ratio, 18, 27, and 7 SNPs on all 12 chromosomes were detected in the whole population, *XI*, and *GJ* panels, respectively, with PVE values of 1.79–5.54%, 1.11–5.49%, and 4.40–8.16%, respectively. Among these associated SNPs, rs10_1541341 was simultaneously detected in the whole population and *XI* panels. Two QTLs, *qCN-5.2* and *qCN-11.1*, detected in the single-locus GWAS were also identified as SNPs rs5_25521042 in the *XI* panel and rs11_25,603,546 in the *GJ* panel in the multi-locus GWAS. However, QTLs/genes related to the C/N ratio were not identified in these two regions, suggesting these regions may contain a potentially novel gene that should be finely mapped (Additional file 5: Table S5).

### Haplotype Analyses for Candidate Genes

A total of 239 annotated genes located in the 16 QTLs detected in the single-locus GWAS underwent a haplotype analysis, and 113 genes were screened as candidate genes (Additional file 6: Table S6). These candidate genes were associated with at least eight plant metabolic pathways in the Kyoto Encyclopedia of Genes and Genomes (KEGG) pathway database (Additional file 6: Table S6), including the flavonoid biosynthesis pathway. Flavonoids reportedly negatively affect starch synthesis in rice (Zhan et al. 2017). Four representative candidate genes were selected for the subsequent comprehensive analysis (Additional file 7: Table S7) according to the intensity of the association signals in the single-locus GWAS, the significance of the haplotype analyses (ANOVA), the biochemically related functions, and the expression profiles.

**Fig. 3:**
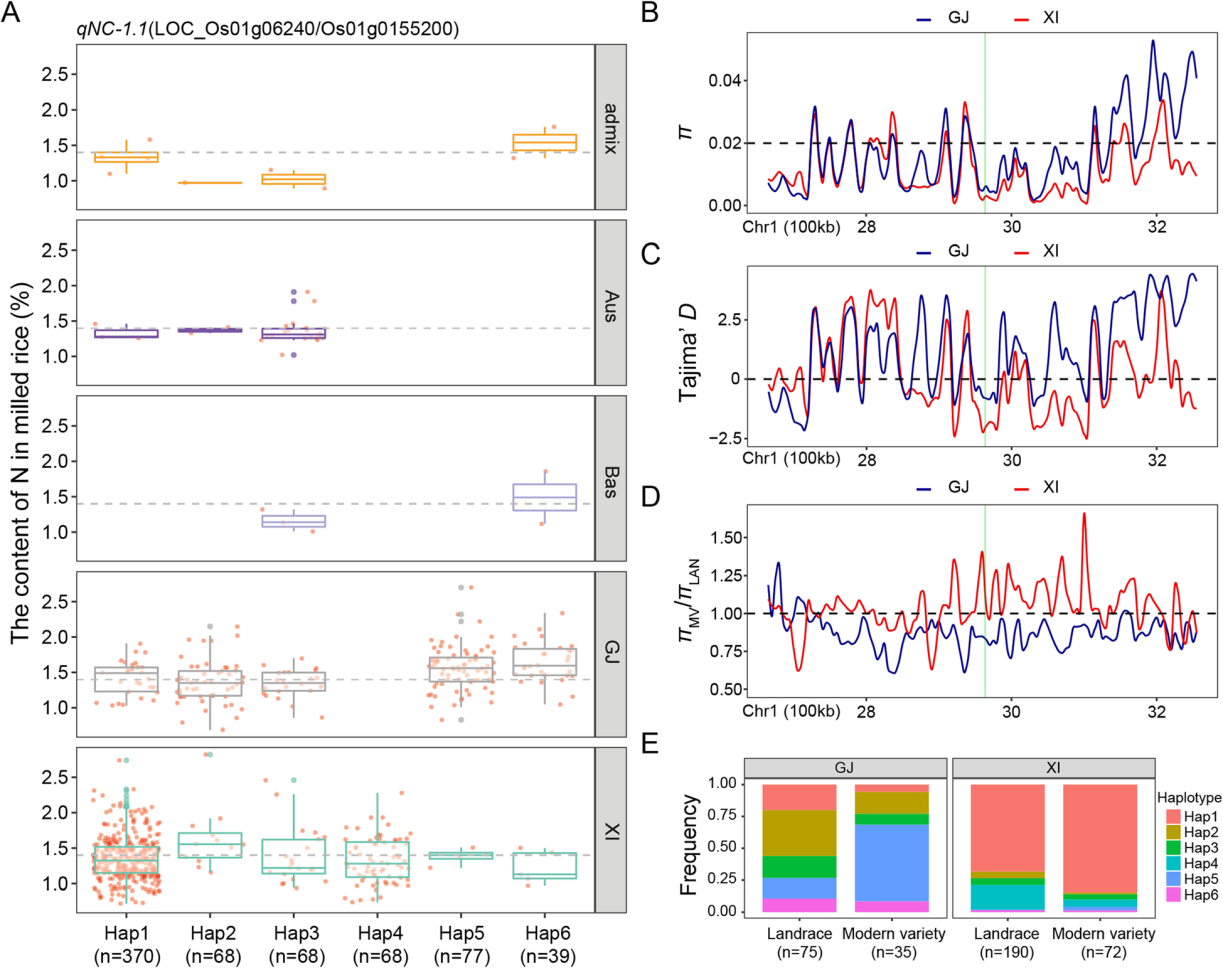
Haplotype analyses for the GNC and nucleotide diversity of the *LOC_Os01g06240* candidate gene at *qNC-1.1*. **a** Haplotype analyses and comparisons of the mean GNC vs *LOC_Os01g06240* haplotypes in five rice subgroups. Each red point around each boxplot indicates the GNC of one accession with the relative haplotype in its subgroup. Haplotypes in fewer than 10 accessions are not shown. **b** Nucleotide diversity (*π*), (**c**) Tajima’s *D* statistics, and (**d**) the ratio of the nucleotide diversity between modern varieties and landraces for the 600-kb genomic region flanking *LOC_Os01g06240*. Red and blue lines represent *XI* and *GJ* subgroups, respectively. The translucent green rectangle represents the *LOC_Os01g06240* genomic region. **e** Frequencies of different haplotypes of *LOC_Os01g06240* in *XI* and *GJ* landraces and modern varieties

For the GNC, six genes annotated based on the Nipponbare reference genome IRGSP 1.0 with at least two haplotypes at *qNC-1.1* (position 2,931,216 to 2,967,102 bp on chromosome 1) were concatenated by SNPs within the gene coding sequence region. Specifically, *LOC_Os01g06240*, which encodes a protein kinase, was detected as the candidate gene with the most significant differences (*P* = 9.80E-08) in the mean GNC among six haplotypes (Additional file 6: Table S6). The frequencies of all six haplotypes were significantly associated with the rice subgroups according to Fisher’s exact tests (Additional file 7: Table S7). Additionally, 94.8 and 76.9% of the accessions with the high-GNC haplotypes Hap5 (*n* = 77) and Hap6 (*n* = 39), respectively, as well as 77.9% of the accessions with the low-GNC haplotype Hap2 (*n* = 68) belonged to the *GJ* subgroup. In contrast, 90.3 and 100% of the accessions with the low-GNC haplotypes Hap1 (*n* = 370) and Hap4 (*n* = 68), respectively, belonged to the *XI* subgroup (Fig. 3a and Additional file 7: Table S7). Moreover, in the *GJ* subgroup, the frequency of Hap5 increased from 0.16 in LAN to 0.60 in MV, whereas the frequencies of the other four haplotypes (Hap1, Hap2, Hap3, and Hap6) were lower in MV than in LAN (Fig. 3). In the *XI* subgroup, the frequency of Hap1 increased slightly from 0.68 in LAN to 0.85 in MV. We analyzed the nucleotide diversity (*π*) and Tajima’s *D* statistics for a 600-kb region flanking *LOC_Os01g06240* in the *XI* and *GJ* subgroups (Fig. 3b, c). The *π*_*GJ*_ and *π*_*XI*_ were similarly lower for the *LOC_Os01g06240* region than for the flanking region, ranging from 0.002 to 0.005 (Fig. 3b). In terms of the allele frequency distributions, Tajima’s *D* was significantly more negative at *LOC_Os01g06240* in the *XI* subgroup than in the *GJ* subgroup (Fig. 3c), implying there is an excess of rare alleles in the *XI* subgroup. Moreover, the *π*_MV_/*π*_LAN_ ratio for *LOC_Os01g06240* was 1.226 and 0.838 in the *XI* and *GJ* subgroups, respectively (Fig. 3d), suggesting *LOC_Os01g06240* may have been affected by selective breeding more in the *GJ* subgroup than in the *XI* subgroup.

**Fig. 4:**
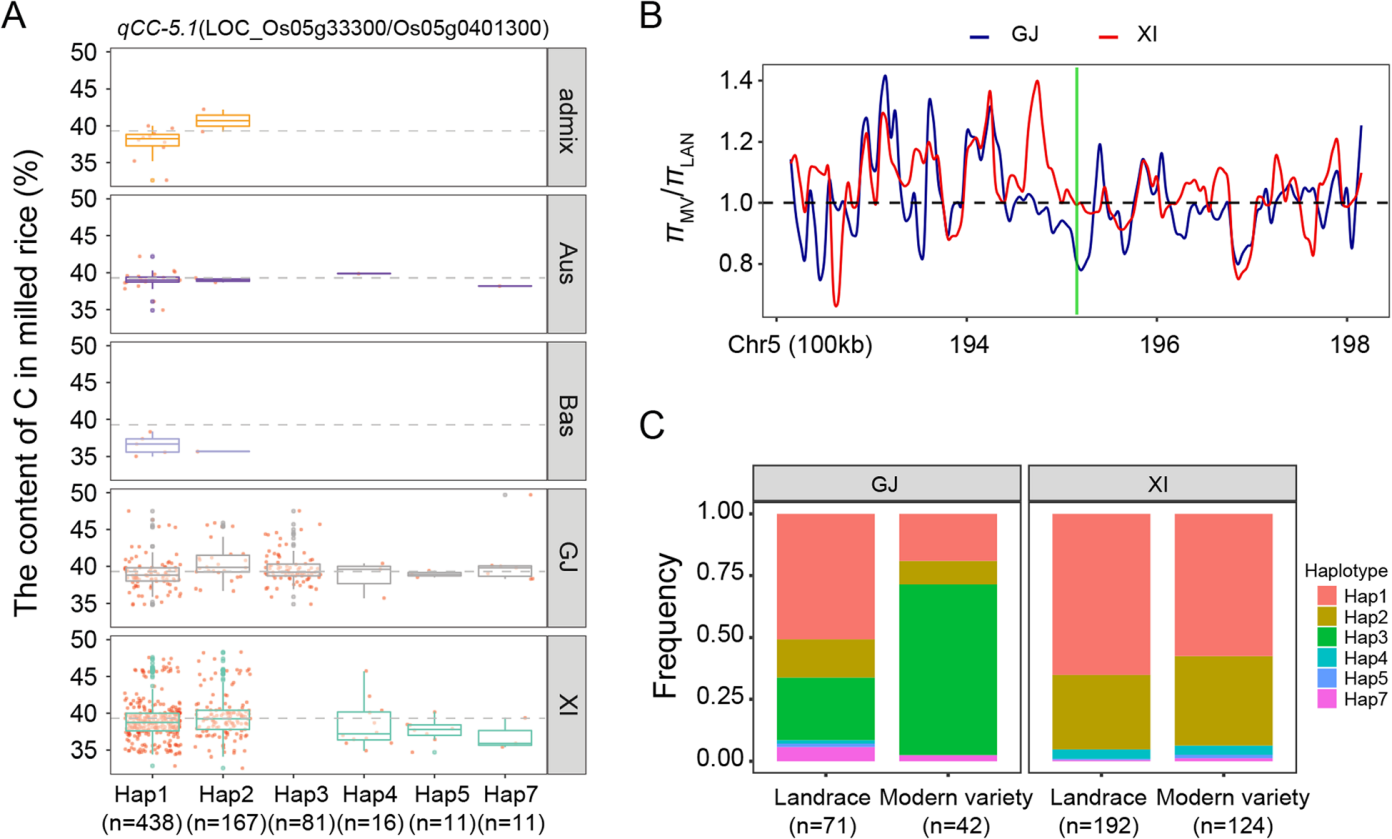
Haplotype analyses for the GCC and nucleotide diversity of the *LOC_Os05g33300* candidate gene at *qCC-5.1*. **a** Haplotype analyses and comparisons of the mean GCC vs *LOC_Os05g33300* haplotypes in five rice subgroups. Each red point around each boxplot indicates the GCC of one accession with the relative haplotype in its subgroup. Haplotypes in fewer than 10 accessions are not shown. **b** Ratio of the nucleotide diversity between modern varieties and landraces for the 600-kb genomic region flanking *LOC_Os05g33300*. Red and blue lines represent *XI* and *GJ* subgroups, respectively. The translucent green rectangle represents the *LOC_Os05g33300* genomic region. **c** Frequencies of different haplotypes of *LOC_Os05g33300* in *XI* and *GJ* landraces and modern varieties

Regarding the GCC, our haplotype analysis revealed four, three, eight, two, and three candidate genes at the *qCC-2.1*, *qCC-2.2*, *qCC-5.1*, *qCC-5.2*, and *qCC-7.1* QTLs, respectively (Additional file 6: Table S6). Additionally, *LOC_Os05g33300*, which encodes a Tat pathway signal sequence family protein, was detected as the candidate gene with the most significant differences (*P* = 4.90E-03) in the mean GCC among six haplotypes carried by at least 10 accessions (Additional file 6: Table S6). Moreover, 81.4 and 100% of the accessions with the representative high-GCC haplotypes Hap2 (*n* = 167) and Hap3 (*n* = 81) belonged to the *XI* and *GJ* subgroups, respectively. The frequency distributions of these two haplotypes differed significantly between the *XI* and *GJ* subgroups (Fig. 4a and Additional file 7: Table S7). Furthermore, the *π*_MV_/*π*_LAN_ ratio for *LOC_Os05g33300* was 0.83 and 1.00 in the *GJ* and *XI* subgroups, respectively (Fig. 4b). In the *XI* subgroup, the frequency of Hap2 increased from 0.30 in LAN to 0.36 in MV, whereas the frequency of Hap1 (low-GCC haplotype) decreased from 0.65 in LAN to 0.58 in MV (Fig. 4c). These results likely partially explain the greater GCC in MV than in LAN in the *XI* subgroup (Fig. 1c).

**Fig. 5:**
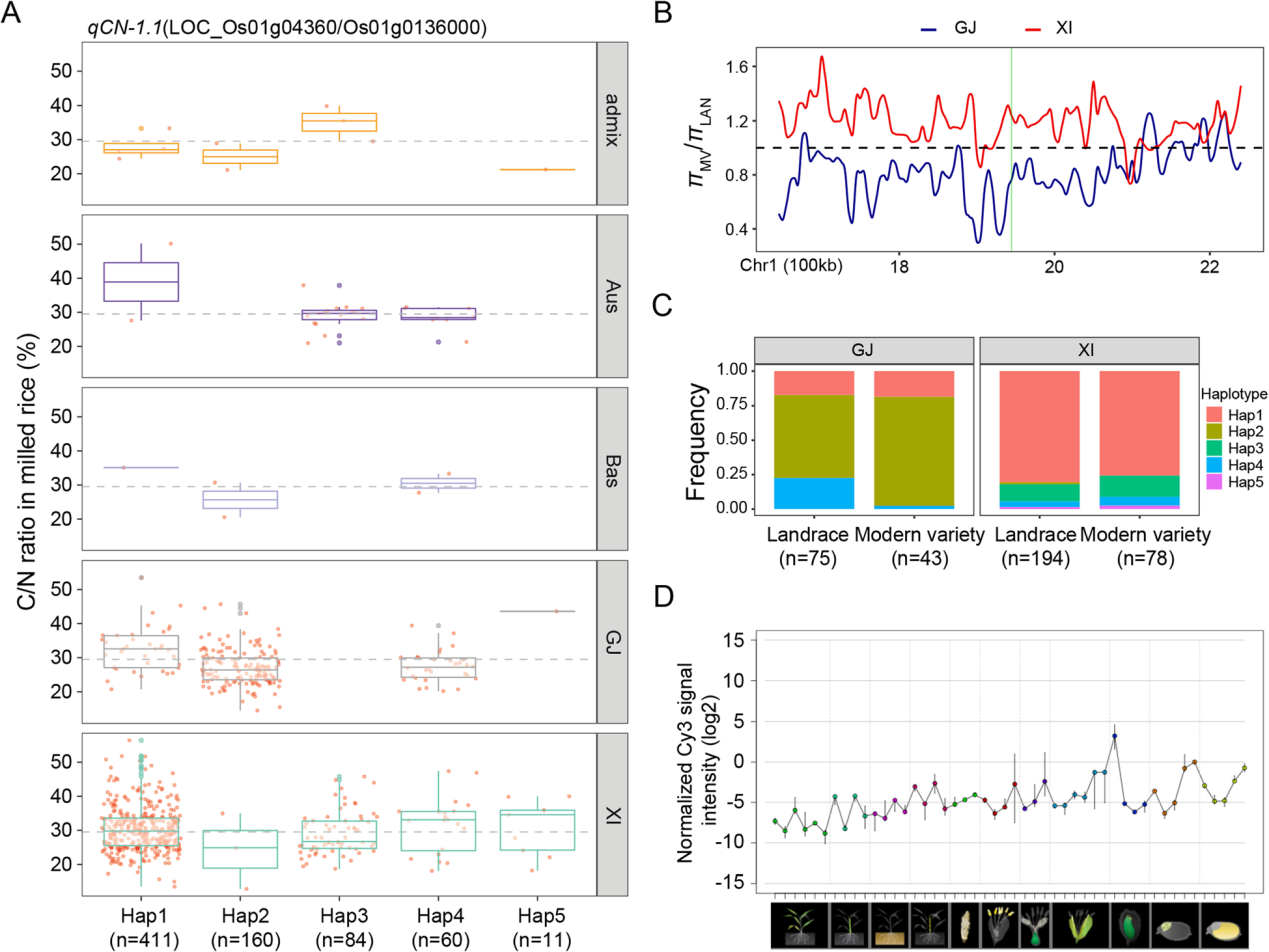
Haplotype analyses for the C/N ratio and nucleotide diversity of the *LOC_Os01g04360* candidate gene at *qCN-1.1*. **a** Haplotype analyses and comparisons of the mean C/N ratio vs *LOC_Os01g04360* haplotypes in five rice subgroups. Each red point around each boxplot indicates the C/N ratio of one accession with the relative haplotype in its subgroup. Haplotypes in fewer than 10 accessions are not shown. **b** Ratio of the nucleotide diversity between modern varieties and landraces for the 600-kb genomic region flanking *LOC_Os01g04360*. Red and blue lines represent *XI* and *GJ* subgroups, respectively. The translucent green rectangle represents the *LOC_Os01g04360* genomic region. **c** Frequencies of different haplotypes of *LOC_Os01g04360* in *XI* and *GJ* landraces and modern varieties. **d** Normalized spatio-temporal expression of *LOC_Os01g04360* in various Nipponbare tissues throughout the entire growth period in the field [downloaded from RiceXPro (version 3.0)]

**Fig. 6:**
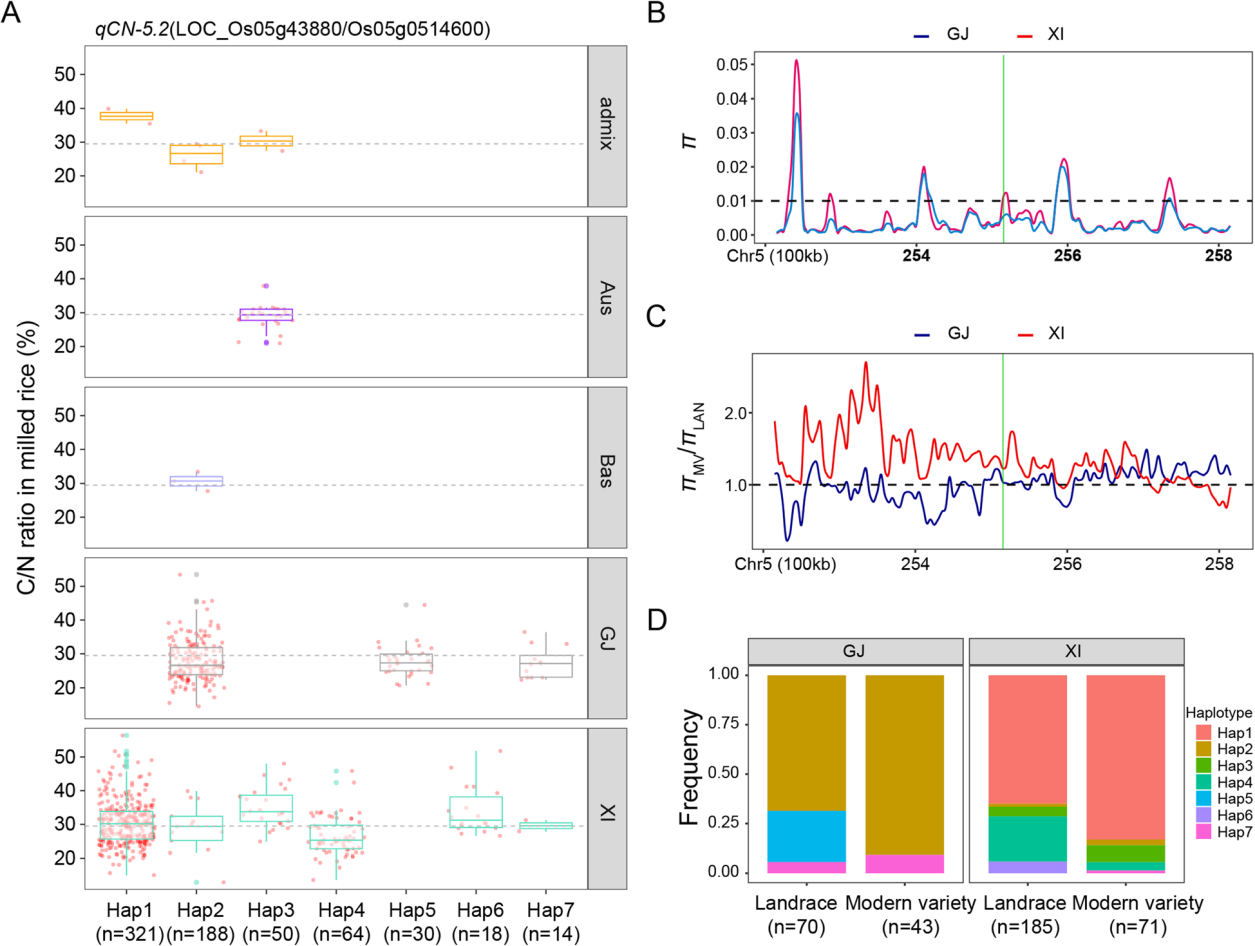
Haplotype analyses for the C/N ratio and nucleotide diversity of the *LOC_Os05g43880* candidate gene at *qCN-5.2*. **a** Haplotype analyses and comparisons of the mean C/N ratio vs *LOC_Os05g43880* haplotypes in five rice subgroups. Each red point around each boxplot indicates the C/N ratio of one accession with the relative haplotype in its subgroup. Haplotypes in fewer than 10 accessions are not shown. **b** Nucleotide diversity (*π*) and (**c**) the ratio of the nucleotide diversity between modern varieties and landraces for the 600-kb genomic region flanking *LOC_Os05g43880*. Red and blue lines represent *XI* and *GJ* subgroups, respectively. The translucent green rectangle represents the *LOC_Os05g43880* genomic region. **d** Frequencies of different haplotypes of *LOC_Os05g43880* in *XI* and *GJ* landraces and modern varieties

For the C/N ratio, 2, 35, 2, 19, 11, 20, 1, and 1 candidate genes were detected at the *qCN-1.1*, *qCN-1.2*, *qCN-3.1*, *qCN-4.1*, *qCN-5.1*, *qCN-5.2*, *qCN-9.1*, and *qCN-11.1* QTLs, respectively, based on our haplotype analysis (Additional file 6: Table S6). Additionally, *LOC_Os01g04360* at *qCN-1.1* and *LOC_Os05g43880* at *qCN-5.2* were screened as important candidate genes with significant differences (*P* = 6.20E-08 and *P* = 6.90E-10) in the mean C/N ratio among different haplotypes in at least 10 accessions (Figs. 5 and 6 and Additional file 6: Table S6). The *LOC_Os01g04360* candidate gene, which encodes a hsp20/alpha crystallin family protein, is highly expressed in specific organs (ovary, embryo, and endosperm) according to a publicly available rice gene expression profile database [RiceXPro (version 3.0)] (Fig. 5d). A comparison of the C/N ratios for the five haplotypes revealed that the representative high-C/N and low-C/N haplotypes were Hap1 and Hap2, with mean C/N ratios of 30.6 and 26.9%, respectively (Additional file 7: Table S7). Moreover, Hap1 and Hap2 were the major haplotypes in the *XI* and *GJ* subgroups, respectively, with significantly different frequency distributions between the two subgroups (Additional file 7: Table S7). We determined that 368 of 411 accessions (89.5%) with Hap1 belonged to the *XI* subgroup, whereas 153 of 160 accessions (95.6%) with Hap2 belonged to the *GJ* subgroup. The *π*_MV_/*π*_LAN_ ratio for *LOC_Os01g04360* was 0.73 and 1.27 in the *GJ* and *XI* subgroups, respectively (Fig. 5b). Furthermore, in the *GJ* subgroup, the frequency of Hap2 increased from 0.60 in LAN to 0.79 in MV, whereas the frequency of Hap4 (relatively low-C/N haplotype) decreased from 0.23 in LAN to 0.02 in MV (Fig. 5c).

The *LOC_Os05g43880* sequence (encoding a gibberellin 2-beta-dioxygenase) was slightly more diverse in the *XI* subgroup than in the *GJ* subgroup (Fig. 6b). The *π*_MV_/*π*_LAN_ ratio for *LOC_Os05g43880* in the *XI* subgroup was a little higher than that in the *GJ* subgroup (Fig. 6c). Multiple comparisons of the C/N ratios for the seven haplotypes indicated the representative high-C/N and low-C/N haplotypes were Hap6 and Hap4, with mean C/N ratios of 34.1 and 26.5%, respectively (Additional file 6: Table S6). The relatively low-C/N haplotypes (Hap2, Hap5, and Hap7) were significantly more abundant in the *GJ* subgroup, whereas the relatively high-C/N haplotypes (Hap1, Hap3, and Hap6) as well as one relatively low-C/N haplotype (Hap4) were mainly detected in the *XI* subgroup (Fig. 6a and Additional file 7: Table S7). These results partially explain the significant differences in the C/N ratio between *XI* and *GJ* (Fig. 1b). Furthermore, in the *GJ* subgroup, the frequency of Hap2 (mean C/N ratio of 28.0%) increased from 0.69 in LAN to 0.91 in MV, whereas the frequency of Hap5 (mean C/N ratio of 27.8%) decreased from 0.23 in LAN to 0 in MV (Fig. 6d), which partially explains the significant differences in the C/N ratio between LAN and MV in this subgroup (Fig. 1c).

## Discussion

### Simultaneous High-Throughput Phenotyping for GNC and GCC

The high-throughput and accurate phenotyping for target traits is currently more important and challenging than the genotyping by next-generation sequencing in a large-scale GWAS. In this study, we used an elemental analyzer based on the Dumas combustion method to rapidly and accurately determine the GNC and GCC in a large population set comprising 751 rice accessions. The application of the Dumas combustion method for analyzing cereals reportedly produces satisfactorily accurate results over a long period (Beljkaš et al. 2010). The repeatability and reproducibility standard deviations for analyses of cereals are lower than required by the Association of Official Analytical Chemists (Beljkaš et al. 2010). Compared with the Kjeldahl method (Beljkaš et al. 2010) for analyzing GPC and the chemical oxidation method (Isabella et al. 2004) for analyzing GCC, the Dumas combustion method is simpler, faster, and produces fewer system errors when simultaneously analyzing the GNC, GCC, and C/N ratio of one sample. Thus, despite the considerable cost and the equipment required for the Dumas combustion method, it is suitable for the high-throughput phenotyping for GNC and GCC in milled rice.

### The GNC and GCC Were Affected by Diverse Selective Breeding in *XI* and *GJ*

Grain quality, which is a complex trait controlled by multiple genes, influences the milling, appearance, eating and cooking qualities, and nutritional qualities of rice. The nutritional quality of rice is mainly affected by the GPC and amino acid composition. Protein is the second most abundant component of rice grains, accounting for 7–10% of the rice endosperm dry weight (Martin and Fitzgerald 2002). The GPC is generally believed to be negatively correlated with the palatability and cooking quality of rice (Ning et al. 2010). A high GPC may lead to densely structured rice grains, which will result in hard and loose cooked rice (i.e., poor palatability) (Martin and Fitzgerald 2002). However, a high GPC will result in rice grains with a high nutritional quality (Long et al. 2013). Thus, the breeding targets for improving rice grain quality largely depend on the food preferences of consumers and the expected end-use of grains in various rice-growing regions worldwide. In this study, the GCC and GNC, which are two fundamental starch and protein characteristics that usually affect rice grain quality, were significantly different among the analyzed rice subgroups, especially between *XI* and *GJ* (Fig. 1b).

The GPC varied considerably between *XI* and *GJ*, with obvious regional differences. There is a high demand for rice varieties with grains that are rich in energy and nutrients among consumers in developing countries (e.g., in South and Southeast Asia), where *XI* varieties are commonly cultivated. In contrast, improving the rice grain quality is increasingly becoming a high priority among consumers in developed countries (e.g., in East Asia), where *GJ* varieties are predominant. In the present study, we revealed that the GNC varied more in the *XI* subgroup than in the *GJ* subgroup (Fig. 1b and Additional file 1: Table S1), which is consistent with the results of earlier studies on GPC (Chen et al. 2018; Zhou et al. 2009). However, the mean GNC of *XI* accessions was significantly lower than that of *GJ* accessions (Fig. 1b), which contradicts the findings of the reported studies on GPC (Chen et al. 2018; Zhou et al. 2009). This discrepancy may be due to the differences in the analyzed sample populations among the studies. Interestingly, the GNC was lower in MV than in LAN in the *GJ* subgroup, but not in the *XI* subgroup, and the GNC in MV was similar between the *XI* and *GJ* subgroups (Fig. 1c). These observations are suggestive of a stronger directional selection for the GNC in *GJ* than in *XI*. In other words, the GNC (or GPC) in modern *GJ* varieties was decreased during breeding to improve eating and cooking qualities.

Rice starch quality, which varies considerably between *GJ* and *XI* accessions, greatly influences rice cooking and processing methods for food and industrial applications (Umemoto et al. 1999). The substantial difference in the resistance to starch disintegration between *GJ* and *XI* is attributed to the diversity in the fine structures of the amylopectin in starch granules (Nakamura et al. 2002). Starch comprises 90% of the total dry weight of milled rice, and the amylose content is considered to be the most important factor affecting eating quality (Pang et al. 2016). In the present study, the GCC was significantly higher in MV than in LAN in the *XI* subgroup, whereas a similar significant difference in the GCC was not detected in the *GJ* subgroup (Fig. 1c). These results imply that increasing grain yield was a greater priority for *XI* varieties than for *GJ* varieties among breeding programs.

### Comparisons with the Previously Reported Genes Related to GPC or GSC

The GPC- and GSC-related known genes near the QTLs identified in this study provide valuable information for thoroughly elucidating the putative genetic mechanisms underlying the GNC and GCC in rice. Although a number of QTLs for the GPC have been identified in rice germplasm (Liu et al. 2010; Ye et al. 2010; Zheng et al. 2011, 2012), relatively few have been cloned. A previous study proved that several mutations in a few genes have minor effects on the GPC and amino acid composition (Kawakatsu et al. 2010a). In the current study, some of the QTLs were only identified in *XI* or *GJ*. For example, a single-locus GWAS revealed four (*qCN-1.2*, *qCN-7.1*, *qCN-9.1*, and *qCN-11.1*) and three (*qCN-3.1*, *qCN-4.1*, and *qCN-5.2*) QTLs for the C/N ratio that were exclusive to the *GJ* and *XI* subgroups, respectively (Table 1). Moreover, *OsAPP6* expression is reportedly associated with GPC variations only in *XI* rice (Peng et al. 2014). A recent study indicated that *OsGluA2*^*LET*^ and *OsGluA2*^*HET*^, which are two *OsGluA2* haplotypes, are present mainly in *GJ* and *XI* varieties, respectively (Yang et al. 2019). Thus, our results provide further evidence that the differences in the GPC between *XI* and *GJ* varieties depend on the diversity in the genetic architecture (Shi et al. 1999).

We searched the Oryzabase online resource (https://shigen.nig.ac.jp/rice/oryzabase/) for known rice genes that co-localized with the 16 QTLs identified in our single-locus GWAS. None of the identified genes are directly related to the GPC or GSC. When we extended the search to regions adjacent to these QTLs (within 100 kb), three known genes (*CCRT*, *OsBZR1*, and *OsPPDKB*) related to the GPC or GSC were detected. Regarding the GCC, *qCC-5.2*, with the most significant associated SNP (rs5_29562689, *P* = 7.43 × 10^− 8^) in the whole population panel, was detected close (approximately 76 kb downstream) to the starch synthesis-related gene *CCRT* (Table 1). A previous study proved that CCRT, which positively regulates starch synthesis in rice vegetative organs, is responsive to the photosynthate content and co-regulates the expression of rice genes related to starch synthesis (Morita et al. 2015). The C/N ratio is significantly and positively correlated with rice grain yield (Ye et al. 2014). In the current study, *qCN-7.1*, with a suggestive association (rs7_23515974, *P* = 2.99 × 10^− 6^) in the *GJ* panel, was detected approximately 30 kb from *OsBZR1*, which encodes a BR-signaling factor. The overexpression of *OsBZR1* can enhance sugar accumulation and increase the grain yield. Knocking down this gene decreases the rice grain weight and starch accumulation. During the pollen and grain development in rice, OsBZR1 can directly promote *CSA* expression, which directly leads to the expression of genes related to sugar distribution and metabolism (Zhu et al. 2015). Additionally, *OsPPDKB*, which encodes a regulator of carbon metabolism (Kang et al. 2005), is located about 56 kb downstream from *qCC-5.1* and *qCN-5.1* (19,481,277–19,681,277 bp) on chromosome 5. Moreover, OsPPDKB regulates the carbon flow associated with starch and fat biosynthesis during the grain-filling period. Compared with the wild-type control, the *floury endosperm-4* mutant generated via the insertion of a T-DNA into *OsPPDKB* has a significantly higher fat content, a slightly higher GPC, and a similar GCC (Kang et al. 2005).

Another three known genes related to the GPC and GCC were detected near the significantly associated SNPs in a multi-locus GWAS. Specifically, *GIF1*, which encodes a cell wall invertase required for carbon partitioning during the early grain-filling period (Wang et al. 2008), is located near the GCC-associated SNP rs4_20223533 in the *GJ* panel. Additionally, *PFPβ*, which regulates carbon metabolism during the rice grain-filling period (Duan et al. 2016), and *OsAlaAT1*, which is essential for the regulation of starch storage in rice endosperm (Yang et al. 2015), were respectively detected near the significant SNPs rs6_7739418 and rs10_13054571 associated with the C/N ratio in the whole panel. These results provide insights into the genetic basis of the variations in the GNC and GCC involving multiple QTLs/genes.

### Utility of the Favorable Haplotypes of Candidate Genes

An apparent strength of the GWAS is that it is convenient for identifying favorable alleles/haplotypes at associated loci in a large set of natural populations and for screening for appropriate germplasm carrying the target alleles/haplotypes for the subsequent breeding of new varieties. According to our results, a method combining a single-locus and a multi-locus GWAS is more powerful than classical bi-parental linkage mapping methods for identifying QTLs for complex traits controlled by multiple genes. The QTLs and the representative haplotypes of the candidate genes described herein (Additional file 6: Table S6) may be useful for future gene cloning and molecular breeding aimed at rapidly improving the GNC and GCC in rice.

The synthesis of starch from sugar (photosynthates/carbohydrates) consumes less energy than the synthesis of other rice grain components, and is conducive to dry-matter accumulation and high yield. Additionally, specific rice-based products, including rice flour (for noodles), rice syrup, and feed rice, require grains that differ in terms of the GSC and GPC. Rice is an important source of nutrition for people and animals (livestock and poultry) in developing countries. Thus, one strategy for breeding high-yielding varieties with a high GPC involves applying marker-assisted selection to pyramid the representative high-GCC, high-GPC, and low-C/N alleles/haplotypes for *LOC_Os01g06240* (Hap6 and Hap5) at *qNC-1.1*, *LOC_Os05g33300* (Hap2 and Hap3) at *qCC-5.1*, *LOC_Os01g04360* (Hap2) at *qCN-1.1*, and *LOC_Os05g43880* (Hap4) at *qCN-5.2* (Figs. 3, 4, 5 and 6 and Additional file 7: Table S7). However, there is also a demand in developed countries for rice varieties with grains that have a relatively low GPC, which enhances the taste. To satisfy this demand, rice breeders should focus on applying representative high-GPC and high-C/N alleles/haplotypes for *LOC_Os01g06240* (Hap4) at *qNC-1.1*, *LOC_Os01g04360* (Hap5 and Hap1) at *qCN-1.1*, and *LOC_Os05g43880* (Hap6 and Hap3) at *qCN-5.2* (Figs. 4, 5 and 6 and Additional file 7: Table S7). Furthermore, the effects of stacking favorable alleles/haplotypes at these loci will need to be investigated.

## Conclusions

The QTLs for the GNC, GCC, and C/N ratio identified in this study may be useful for clarifying the molecular mechanism underlying the GNC and GCC. Our findings may also be relevant for enhancing the application of the favorable haplotypes of candidate genes during the molecular breeding of new rice varieties that satisfy the diverse demands for the GNC and GCC.

## Methods

### Rice Germplasm and Evaluation of the GNC and GCC in Milled Rice

The GNC and GCC of the milled rice grains of 751 accessions with appropriate and similar heading dates from the 3K RGP (3K RGP 2014) (Additional file 1: Table S1) were evaluated. On the basis of the known population structure and division of subpopulations (Wang et al. 2018), the 751 accessions comprised 475 *XI* accessions (73 *XI-1A*, 29 *XI-1B*, 74 *XI-2*, 113 *XI-3*, and 186 *XI-adm*), 231 *GJ* accessions (136 *GJ-tmp*, 35 *GJ-sbtrp*, 35 *GJ-trp*, and 25 *GJ-adm*), 27 *Aus* accessions, 6 *Bas* accessions, and 12 admixture (*adm*) accessions. Accessions were planted in Sanya, China in 2018 and 28-day-old seedlings were transplanted to field plots with three rows of eight plants (20 × 17 cm spacing) for each accession. Two replicates were prepared for each accession. The management of the field plots followed normal local agricultural practices.

At maturity, five plants in the middle of the second row were harvested and bulked for each replicate of every accession. The grains of each accession were threshed and air-dried in a greenhouse. When the moisture content of the grains reached 13%, the samples were prepared for the subsequent analyses as follows. After milling and crushing, the grain samples were passed through a 100-mesh sieve, after which 80 mg rice flour was placed in a tin paper cylinder, wrapped, and pressed to form a medicinal tablet shape. The GNC and GCC of the milled rice samples were analyzed with the vario MACRO cube (Elementar Co., Hanau, Germany), which is based on the Dumas combustion method. During the measurements, the working temperatures of the combustion tube and reduction tube were set at 1150 °C and 850 °C in the CNS mode, respectively. The helium intake pressure was set at 1200–1250 mbar and the flow rate was approximately 600 ml/min. The mean trait values for the two replicates were used for the GWAS.

### Statistical Analyses of Phenotypic Data

Differences in the mean GNC, GCC, and C/N ratio among the rice subgroups were evaluated by a one-way ANOVA and Duncan’s multiple mean comparison test (5% significance level), which were completed with the agricolae package in R. Correlation analyses of the three traits were conducted with the corrplot package in R.

### Single-Locus GWAS

The 3K RGP 4.8mio SNP dataset was downloaded from the Rice SNP-Seek Database (http://snp-seek.irri.org/) (Alexandrov et al. 2015). The 2,994,907, 2,118,326, and 1,318,493 SNPs with minor allele frequencies > 5% and a missing data rate < 0.1 filtered by PLINK (Purcell et al. 2007) for the whole population, *XI*, and *GJ* panels, respectively, were used for the subsequent association analyses (Additional file 3: Table S3). The single-locus GWAS was completed with EMMAX (Kang et al. 2010) to determine the associations between each SNP and the GNC, GCC, and C/N ratio of milled rice. A Balding–Nichols matrix based on the pruned subset of genome-wide SNP data (with the ‘indep-pairwise 50 10 0.1’ parameter in PLINK) was used to create the kinship matrix. We calculated the eigenvectors of the kinship matrix with GCTA (Yang et al. 2011) and then used the first three principal components as covariates to capture the variance due to the population structure. The effective number of independent markers (*N*) was calculated with the GEC software (Li et al. 2012) and suggestive *P*-value thresholds of association (1/N) were calculated (Additional file 3: Table S3). We identified the genes harboring or flanking the suggestively associated SNPs and functionally annotated them based on the Nipponbare reference genome IRGSP 1.0 (Kawahara et al. 2013). The Manhattan and quantile-quantile plots for the GWAS results were created with the R package qqman (Turner 2014). To detect independently associated regions, multiple suggestively associated SNPs located in one estimated LD block were clustered as one QTL region, and the SNP with the minimum *P* value in a cluster was considered as the lead SNP. Each LD block containing the detected SNPs was estimated with the ‘--blocks’ command in PLINK according to the block definition suggested by Gabriel et al. (2002).

### Multi-Locus GWAS

The multi-locus GWAS was completed with the same genotypes and phenotypes used for the single-locus GWAS and the multi-locus random-SNP-effect mixed linear model (mrMLM) (Wang et al. 2016) of the mrMLM package (https://cran.r-project.org/web/packages/mrMLM/index.html) in R. A critical LOD score of 3.0 was used for identifying significantly associated SNPs.

### Haplotype Analysis of Candidate Genes

The haplotypes of all 239 genes annotated based on the Nipponbare reference genome IRGSP 1.0 (Kawahara et al. 2013) and located within the 16 detected QTLs in the single-locus GWAS were classified according to all SNPs within the coding sequence region of one gene in the 751 rice accessions. The KEGG pathways associated with these genes were determined with EXPath 2.0 (Chien et al. 2015). Haplotypes in at least 10 rice accessions were used for a phenotypic comparative analysis. A one-way ANOVA followed by Duncan’s test were completed with the agricolae package in R to screen for candidate genes. Four representative candidate genes were selected for a comprehensive analysis based on the intensity of the association signals in the single-locus GWAS, the significance of the haplotype analyses (ANOVA), the biochemically related functions, and the expression profiles. Two-sided Fisher’s exact tests in R were used to compare haplotype frequencies between the rice *XI* and *GJ* subgroups. Nucleotide diversity (*π*) and Tajima’s *D* value for each 10-kb window across the genome, with an overlapping 5-kb step size, were calculated for the 600-kb region flanking the candidate genes with the Variscan program (version 2.0.3) (Vilella et al. 2005). Gene expression profiles were downloaded from a rice expression profile database [RiceXPro (version 3.0)] (Sato et al. 2013).

## Supplementary information

**Additional file 1 **: **Table S1.** Summary of 751 rice accessions and the GNC, GCC, and C/N ratio of the milled rice

**Additional file 2 **: **Table S2.** Correlations among traits observed in different rice subgroups

**Additional file 3 **: **Table S3.** Filtered and effective number of single nucleotide polymorphisms in each GWAS panel and adjusted significant *P*-value thresholds based on a Bonferroni correction

**Additional file 4 **: **Table S4.** List of 55 significant SNPs associated with the GNC, GCC, and C/N ratio of milled rice detected in a single-locus GWAS

**Additional file 5 **: **Table S5.** Association signals detected in a multi-locus GWAS for the GNC, GCC, and C/N ratio of milled rice

**Additional file 6 **: **Table S6.** Comparison of the GNC, GCC, and C/N ratio among the haplotypes of 239 annotated genes within 16 QTLs detected in a single-locus GWAS

**Additional file 7 **: **Table S7.** Haplotype analysis of four representative candidate genes at four QTLs associated with the GNC, GCC, and C/N ratio of milled rice

## Data Availability

All data supporting the conclusions of this article are provided within the article (and in the Additional files).

## Abbreviations

3K RGP: 3000 Rice Genomes Project
*adm*: Admixture
*Bas*: Basmati
BE: Starch branching enzyme
C: Carbon
*GJ*: *Geng*/*Japonica*
GNC: Grain N content
GPC: Grain protein content
GSC: Grain starch content
GWAS: Genome-wide association study
LAN: Landraces
LD: Linkage disequilibrium
MV: Modern varieties
N: Nitrogen
PVE: Phenotypic variation explained
QTL: Quantitative trait locus
SNP: Single nucleotide polymorphism
SS: Soluble starch synthase
SSP: Seed storage protein
*XI*: *Xian*/*Indica*
*π*: Nucleotide diversity
